# Senescent stromal cells induce cancer cell migration via inhibition of RhoA/ROCK/myosin-based cell contractility

**DOI:** 10.18632/oncotarget.5854

**Published:** 2015-10-01

**Authors:** Ivie Aifuwa, Anjil Giri, Nick Longe, Sang Hyuk Lee, Steven S. An, Denis Wirtz

**Affiliations:** ^1^ Johns Hopkins Physical Sciences - Oncology Center, The Johns Hopkins University, Baltimore, Maryland, USA; ^2^ Department of Chemical and Biomolecular Engineering, The Johns Hopkins University, Baltimore, Maryland, USA; ^3^ Departments of Pathology and Oncology, Sydney Kimmel Comprehensive Cancer Center, The Johns Hopkins School of Medicine, Baltimore, Maryland, USA; ^4^ Department of Environmental Health Sciences, Johns Hopkins Bloomberg School of Public Health, Baltimore, Maryland, USA

**Keywords:** SASP, senescence

## Abstract

Cells induced into senescence exhibit a marked increase in the secretion of pro-inflammatory cytokines termed senescence-associated secretory phenotype (SASP). Here we report that SASP from senescent stromal fibroblasts promote spontaneous morphological changes accompanied by an aggressive migratory behavior in originally non-motile human breast cancer cells. This phenotypic switch is coordinated, in space and time, by a dramatic reorganization of the actin and microtubule filament networks, a discrete polarization of EB1 comets, and an unconventional front-to-back inversion of nucleus-MTOC polarity. SASP-induced morphological/migratory changes are critically dependent on microtubule integrity and dynamics, and are coordinated by the inhibition of RhoA and cell contractility. RhoA/ROCK inhibition reduces focal adhesions and traction forces, while promoting a novel gliding mode of migration.

## INTRODUCTION

Advanced age is the main risk factor for a range of human pathologies, including cardiovascular, neurodegenerative and neoplastic syndromes. Ageing involves the accumulation of damage to macromolecules (i.e. nuclear DNA damage, misfolded proteins, and mitochondrial DNA deletions). Ageing-induced damage leads to the malfunction of organelles and promotes cellular defects and tissue dysfunction [1–3]. Age-related pathologies and deficits are, in part, a result of the accumulation of senescent cells in the aging organism [1, 4]. Cellular senescence is a state of irreversible growth arrest that occurs as a result of oncogenic stimulus or genomic stress. This includes telomere erosion, DNA double-strand breaks, genetic defects and overexpression of oncogenes, such as *KRAS* [4, 5]. Growth arrest prevents the perpetuation of cellular damage from one generation to the next and thus provides a potent tumor-suppressive mechanism to cells exposed to oncogenic stimuli.

Despite their anti-tumorigenicity, senescent cells can contribute to neoplastic progression by promoting a pro-inflammatory environment. Transcriptional changes that accompany senescence promote a robust increase in mRNA, translation and the secretion of cytokines, chemokines, growth factors and proteases [4–6]. This complex senescence-associated secretory phenotype (SASP) promotes tissue remodeling and stimulates a malignant phenotype and tumor progression in neighboring epithelial cells. In particular, this pro-inflammatory stimulus elicits aggressive cancer behavior, including enhanced invasion, proliferation, loss of cell-to-cell contacts and an apparent epithelial-mesenchymal transition (EMT) [5, 7–10]. The molecular mechanism underlying this aggressive tumor cell behavior, in particular a transition from a non-motile to motile phenotype, remains largely unknown.

Here, we showed that factors secreted by senescent stromal fibroblasts promote a dramatic morphological change in otherwise round, non-motile cancer cells. This morphological change is accompanied by a strong migratory phenotype in originally non-motile human breast cancer cells. The SASP-induced morphological/migratory switch is associated with a dramatic reorganization of both F-actin and microtubule cytoskeletal networks. Such transitions from a non-motile-to-motile phenotype feature little to no lamellipodial protrusions. Most strikingly, SASP-induced local cellular migrations feature microtubule-enriched tails trailing the migrating cell, with significantly reduced actin assembly along the cell edges. SASP-stimulated cells also display a non-uniform spatial redistribution of microtubule-terminating EB1 comets. Paradoxically, migrating cells conformed to an unconventional inverse, front-to-back polarity of their nucleus and microtubule-organizing center (MTOC); the nucleus is located at the leading migratory front of the cell instead of conventional nuclear positioning at the trailing edge of the cell. This SASP-induced phenotypic switch is mediated by microtubule integrity and dynamics, as well as the inhibition of Rho/ROCK/myosin mediated cell contractility. We demonstrate that Rho inhibition is both necessary and sufficient to initiate and maintain the SASP-induced morphological and migratory behavior of cancer cells. SASP-induced inhibition of RhoA reduces the size and number of focal adhesions and diminishes traction forces, inducing a gliding mode of migration.

## RESULTS

### SASP-induced change in cell morphology is accompanied by onset of migration

To induce cellular senescence, human lung (WI-38) fibroblasts, were treated with bleomycin and allowed to recover for 8 days. Proliferation status of fibroblasts was verified by Ki-67 staining (Figure S1, a and b) and by directly assessing cell doubling (Figure S1c). WI-38 cells developed senescent associated heterochromatic foci observed with phosphorylated H2A.X staining (Figure S1d-g). Cells also drastically increase their cell size, a key morphological feature of senescence (Figure S1h). Cellular senescence induced by bleomycin was accompanied by a robust senescence-associated secretory phenotype (SASP), including elevated levels of interleukins IL-6 and IL-8 (Figure S1, i and j) [11, 12].

**Figure 1:**
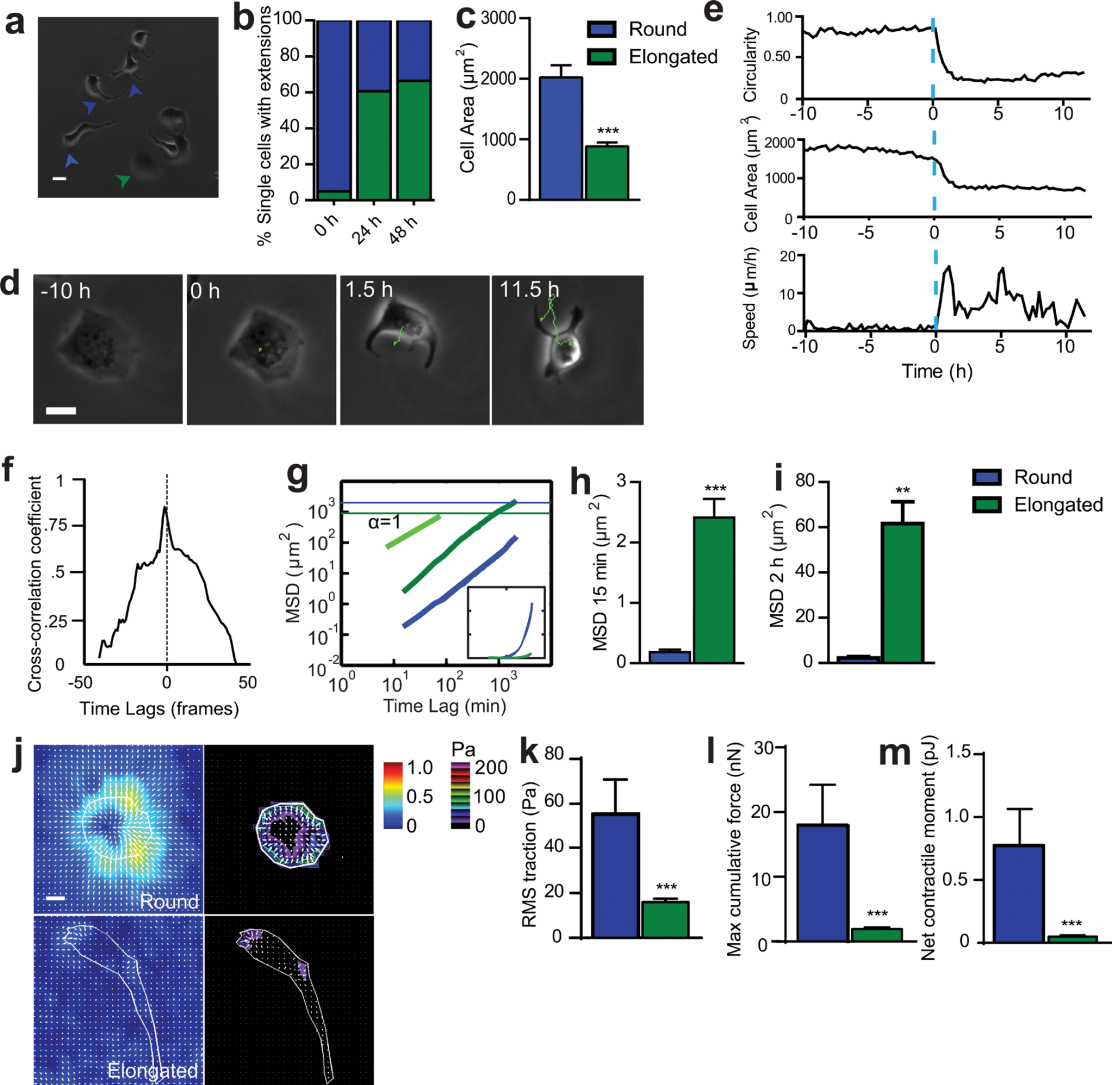
SASP-induced change in cell morphology is accompanied by onset of migration **a.** Phase-contrast micrograph of cells exposed to Sen CM for 48h. Green and blue arrowheads point to cells that became elongated and cells that remained round, respectively. Scale bar, 20μm. **b.** Fraction of elongated (protruded) cells (green) and cells that remained round (blue), assessed 24h and 48h after exposure to Sen CM. Numbers of examined cells are *n* = 62 at 0 h, *n* = 126 at 24h and *n* = 147 at 48 h. **c.** Averaged size of cells exposed to Sen CM. **d.** Typical movie (21.5 h) of a T47D cell undergoing a drastic change in morphology (designated time 0) induced by Sen CM added at time −10h. Scale bar, 20 μm. **e.** Changes in speed, circularity, cell area for the cell shown in **d.**. The blue dotted line indicates time = 0, time at which the cell spontaneously changed morphology, ∼10 h after SASP exposure. **f.** Temporal cross-correlation between cell circularity and cell speed for cell shown in **d.**. Dashed line indicate time lag = 0, which shows the point where events are perfectly synchronized. **g.** Population-averaged mean squared displacement (MSD) of elongated and round T47D cells as a function of time lag. Numbers of examined cells are *n* = 94, over 3 independent experiments. Green and blue horizontal lines are the averaged sizes of elongated cells and round cells, respectively, and indicate that only elongated translocated more than their size over the 36 h of cell tracking. Inset is a linear representation of panel g. **h.** and **i.** Population-averaged MSD evaluated at time lags of 15 min **f.** and 2 h **g.**. Number of examined cells are *n* = 94, over 3 independent experiments. **j.** Bead displacement and traction field of round and elongated cells placed on a 1300 Pa compliant polyacrylamide gel and exposed to Sen CM. Scale bar, 10 μm. (k-m) RMS traction stresses **k.** Maximum cumulative force **l.** and Net contractile moment **m.**from elongated and round cells exposed to Sen CM. Number of examined cells is *n* = 25 *P < 0.05, **P < 0.01 and ***P < 0.001.

To determine whether SASP endowed cancer cells with an aggressive behavior, non-motile, T47D human epithelial breast cancer cells were exposed to conditioned medium from senescent cells (Sen CM). We note that both WI-38 and T47D are standard cell lines used extensively to study the interplay between senescence of fibroblasts and cancer [5, 7, 13]. As previously observed, Sen CM promoted loss of cell-to-cell contact [5]. However, stimulating cells with Sen CM caused a dramatic change in cell morphology, from initially rounded and large to elongated and small in size (Figure 1a, 1c and 1d, Movie S1b). Cells typically featured 1 to 3 long and thick extensions projected towards the back or the sides of the cell (Figure 1a and 1d). Before Sen CM was added, less than 5% of T47D cells displayed an elongated morphology. However, 24h and 48h after exposure to Sen CM, the fraction of cells presenting an elongated morphology increased to 61% and 67%, respectively (Figure 1b). SASP-induced elongated morphology and triggered migration were also observed with T47D cells exposed to Sen CM from BJ human skin fibroblasts and IMR-90 human lung fibroblasts, suggesting that this is not a cell-specific effect that is unique to WI-38 cells (Figure S2).

We next investigated the functional consequences of this drastic morphological switch. The SASP-induced morphological change was accompanied by the onset of cell migration (Figure 1d–1e and Movie S1c). The transition from a rounded, non-motile phenotype to an elongated, motile phenotype occurred within minutes in the presence of Sen CM. To quantify the dynamic change in cell morphology and migration, we simultaneously measured the instantaneous cell speed and time-dependent cell morphological parameters, including cell circularity and cell size (Figure 1e). When cellular circularity and area changed abruptly (blue dotted line), cell speed changed almost simultaneously. This was further demonstrated by analysis of the temporal cross-correlation between cell circularity and speed with the greatest correlation observed at a time lag of almost 0 (i.e., morphological change and onset of migration coincided) (Figure 1f).

The mean square displacement (MSD) of cells computed from the coordinates of the centroids of cells is an effective means to quantify cell migration. We compared the MSDs of cells that changed their morphology and became elongated to cells that maintained their original morphology (circular and round) in response to Sen CM (Figure 1g). Horizontal lines in Figure 1g indicate the averaged cell area of each cell type; they indicate that only elongated cells migrated more than their average size during imaging. Elongated cells displayed significantly higher MSDs than cells that remained round over all probed time lags, indicating that only cells that underwent a round-to-elongated morphological switch in response to SASP could migrate and translocate (Figure 1h and 1i). Migration was also verified using a 3D spheroid assay. Within 24 h exposure to Sen CM (compared to FM), an aggregate of cells embedded in collagen I disseminated into their surrounding collagen network (Figure S2, b and c).

For typical mesenchymal cells, migration requires cell-induced traction forces exerted on the underlying substratum, as cells attach to form new adhesions at the leading edge and detach to disassemble adhesions at the rear of the cell [14–16]. To determine the traction forces exerted by cells exposed to SASP, cells were plated on an inert elastic gel containing 0.2μm-diameter fluorescent beads. Round cells significantly displaced the embedded fluorescent beads and isotropically produced traction forces onto underlying substrate (Figure 1j). Elongated cells, however, generated significantly reduced traction forces (Figure 1j). Traction forces were mainly confined to the leading edge of the cell. Computed traction (root mean square) averaged over the entire cell projected area, maximum cumulative force, and net contractile moment (a scalar measure of cell's contractile strength) were remarkably greater in round cells than elongated cells (Figure 1k–1m). Accordingly, motile, elongated cells displayed significantly decreased contractility compared to their round, non-motile counterparts.

### SASP-induces cytoskeletal reorganization and MTOC- nucleus polarization

Cell morphology and motility are typically controlled by the cytoskeleton, a dense network of filamentous proteins, including intermediate filaments, actin, and microtubules [16, 17]. The roles of the cytoskeleton in SASP-induced morphological change and the onset of migration are unknown. Traditionally, filamentous actin (F-actin) and the associated F-actin-bundling and motor protein myosin II produce the required forces for migration and shape changes accompanying cell migration [16, 18]. In contrast, microtubules, which stem from the microtubule-organizing center (MTOC or centrosome), set the polarization axis of cells. The MTOC is positioned at the front of the cell, while the nucleus is positioned at the back [16, 18].

**Figure 2:**
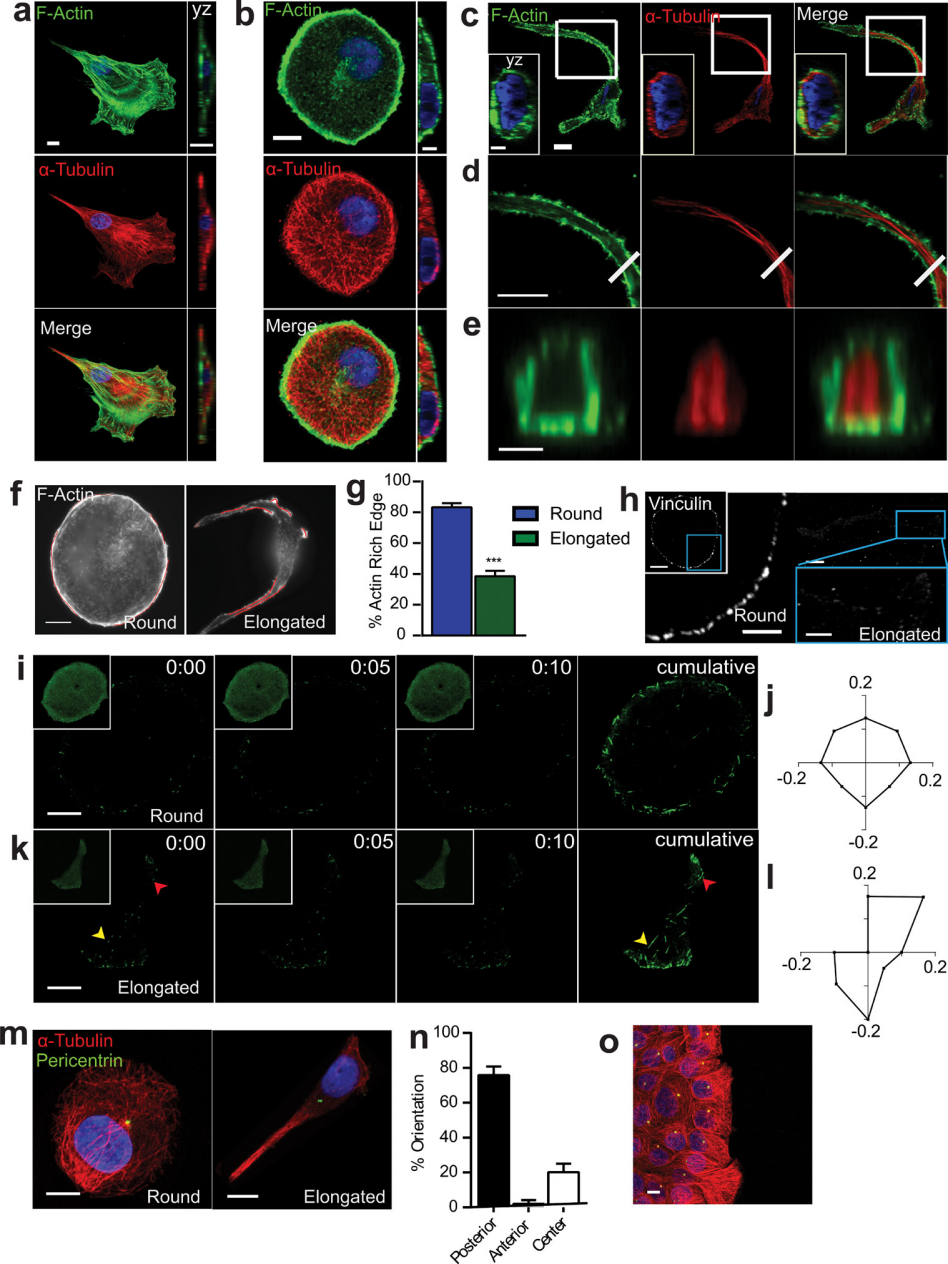
SASP-induced morphological change is accompanied by major cytoskeletal reorganization **a.**-**e.** Fluorescence confocal micrographs of WI-38 fibroblasts **a.**, and round **b.** and elongated **c.** T47D cells stained for F-actin (green), α-tubulin (red) and nuclear DNA (blue) after exposure to Sen CM for 48h. Top xy (first panel) and crossectional yz views (second panel **b.** and inset **c.**). Scale bar, 10 μm. White square indicates the region of T47D cell expanded in **d.**. Panel d displays cytoskeletal filaments of the cell tail in first panel. White line in second panel specifies cross-sectioned region shown in **e.**. Scale bar, 5μm. Panel e displays yz cross section of the elongated T47D cell in first panel. Scale bar, 2 μm. **f.** Fluorescence micrographs of round and elongated cells stained for F-actin with actin-rich regions delineated using polylines, respectively. Scale bar, 10 μm. **g.** Percentage of actin-rich edges on round and elongated cells; *n* = 30. **h.** Fluorescence confocal micrographs of round and elongated cells stained for vinculin, where blue boxes indicate zoomed region of T47D cells. Scale bar, 10 μm in original image and 5 μm in zoomed image. **i.**-**k.** Round **i.** and elongated **k.** T47D cells transfected with EB1-EGFP then exposed to Sen CM for 24h are displayed every 5s for 10s for first 3 panels with a cumulative image shown in the last panel. Larger images optimize the contrast to enhance EB1 comet visibility. Original images are displayed as inset per image. Arrowheads indicate starting and ending points of EB1-EGFP comets within the 10s imaging time. Scale bar, 10 μm. **j.** and **l.** Angular distributions of EB1-EGFP comets within round **j.** and elongated **l.** cells within both cell center and periphery. *n* = 30 comets per cell. **m.** Round (first panel) and elongated (second panel) T47D cells immunostained for α-tubulin (red), pericentrin (green), and nuclear DNA (blue) after exposure to Sen CM for 48h. Scale bar, 10 μm. **n.** Percentage of cells having posterior, anterior and centered MTOC orientation. **o.** Confocal micrograph of T47D cells 48h after scratch wound. Cells were immunostained for α-tubulin (red), pericentrin (green), and nuclear DNA (blue). Scale bar, 20 μm.

An important feature of SASP-induced migration was that cells displayed non-standard cytoskeletal organization. In typical migratory mesenchymal cells such as fibroblasts and fibrosarcoma cells, microtubules are mainly organized throughout the cell body, while actin filaments form a dendritic network in the lamellipodium and stress fibers at the basal surface (Figure 2a). Three-dimensional confocal fluorescence images were collected to visualize the cytoskeletal structures of cells exposed to Sen CM (Figure 2b and 2c). Round cells displayed a dense F-actin meshwork along the cell cortex, with punctate structures within the cell body. Microtubules were organized in dense networks in the cell body and at the cell periphery (Figure 2b). In striking contrast, elongated cells displayed punctate actin in the cortical region of the cell, with significantly reduced actin-rich edges (Figure 2c–2g and Table 1). Within the elongated cell, microtubules formed thin disorganized structures near the nucleus and thick linear bundles in the cell tails (Figure 2c and 2d). Cross-sectional views of cell tails revealed that F-actin and microtubules were spatially segregated: actin filaments formed a corona and bundled microtubule polymers were located in the core (Figure 2d and 2e). A similar morphological changed occurred when MCF7 cells were exposed to Sen CM (Figure S3a). T47D cells exposed to Sen CM from BJ and IMR-90 fibroblasts also underwent the same cytoskeletal changes, indicating that cytoskeletal reorganization was independent of the cells from which Sen CM was obtained (Figure S3, b and c). Consistent with reduced traction forces (Figure 1k), vinculin staining revealed a decrease in focal adhesions in elongated cells compared to round cells, which displayed larger focal adhesions along the cell cortex (Figure 2h).

**Table 1:** Conventional mesenchymal and SASP-induced gliding migration

Conventional mesenchymal miration	SASP-induced gliding migration
Wide lamella and lamellipodium at leading edge	No or little lamella; cell "trails" at trailing edge
Lamella containing few microtubules and a dense acto-myosin-rich cortex	Trails containing a core of microtubule bundles and a dense corona of F-actin
Nucleus at the back of the cell	Nucleus at the front of the cell
MTOC towards leading edge	MTOC towards trailing edge
Activation of Rho/ROCK	De-activation of Rho/ROCK
Increased focal adhesions	Decreased focal adhesions
Increased traction forces	Decreased traction forces
Motility depends weakly on MT assembly dynamics	Migration critically depends on MT assembly/dynamics

In addition to changes in microtubule organization in response to SASP stimulation, we determined next whether microtubule dynamics was also altered. Microtubule dynamics is typically assessed by quantifying the dynamics of EB1, a microtubule plus-end protein (+TIP) that tracks the dynamic assembly of microtubules [19]. Cells were transfected with EB1-EGFP, exposed to Sen CM, and imaged by live-cell confocal microscopy. Microtubule assembly dynamics, as measured by tracking EB1 comets, was similar in round and elongated cells (Figure S2d). Individual images collected 1s apart for 10s were overlay to display the cumulative directionality of EB1 comets. As expected, EB1 comets in round cells showed isotropic directionality (Figure 2i and Movie S2a). In contrast, elongated cells showed polarized EB1 comets (Figure 2k and Movie S2b). EB1 comets were enriched in the cell front and extensions, and disorganized within the cell body. In cells having multiple extensions, comets were enriched and directional in each extension, and comets showed no preferred directionality within the cell body (Figure S2e and Movie S2c). The directionality of actively growing EB1 comets, further quantified by computing their time-dependent angular distributions, indicated that elongated cells had EB1 growth mainly in the polarized direction of the moving cell (Figure 2j and 2l).

Another unique feature of SASP-induced cell migration was that originally non-polarized cells that were set in motion adopted an inverse, “front-to-back” polarization in response to SASP stimulation (Figure 2m–2n, Figure S3f and Table 1). Here, migrating cells positioned their nucleus in the very front of the cell, while the MTOC was located behind the nucleus towards the back of the cell, where front and back are defined by the direction of cell migration. Importantly, this mode of polarization differed from the same cells stimulated to migrate with a scratch-wound. Here, cells oriented their MTOC toward the direction of migration with the nucleus in the back (Figure 2o), which the conventional positions of MTOC and nucleus in many types of migratory cells [20, 21]. Hence, this MTOC-nucleus inverse polarization was not an intrinsic property of these cells, but rather a universal response to SASP stimulation. We note that SASP-exposed round and elongated cells did not feature significant differences in either the distance between the MTOC and nuclear surface or the distance between the nucleus and MTOC centroid (Figure S2, g and h).

### Role of microtubule integrity and dynamics in SASP-induced morphology and migration

**Figure 3:**
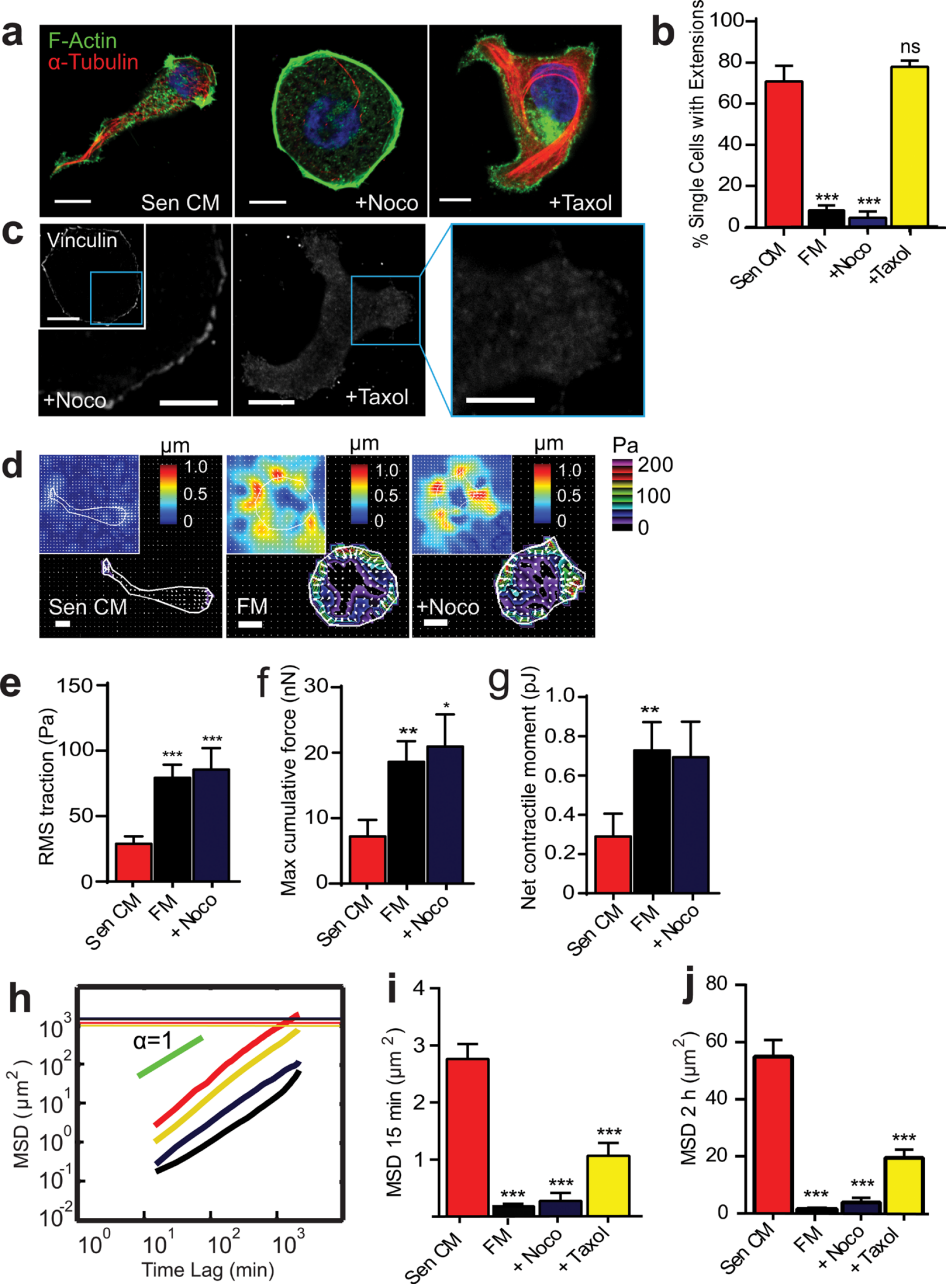
Microtubule integrity and dynamics are required for SASP-induced migration and change in morphology **a.** Fluorescence confocal micrographs of T47D cells after 48h exposure to Sen CM, Sen CM + 0.5 μg/ml nocodazole (Noco) or Sen CM+ 1 μM taxol, respectively, for 48h. Cells are stained for F-actin (green), α-tubulin (red) and nuclear DNA (blue). Scale bar, 10 μm. **b.** Percentage of single cells with extensions when exposed to Sen CM (*n* = 147), Fresh media (FM) (*n* = 57), Sen CM+Noco (*n* = 50), Sen CM+Taxol (*n* = 54), respectively, after 48h. *P* = 0.1801for Sen+Taxol **c.** Fluorescence confocal micrographs of cells exposed to Sen CM+Noco and Sen CM+Taxol stained for vinculin, where blue boxes indicate zoomed region of T47D cells. Scale bar, 10 μm in original image and 5 μm in zoomed image. **d.** Bead displacement and traction field of T47D cells placed on a 1300 Pa compliant polyacrylamide gel and exposed to Sen CM (*n* = 25), SF (*n* = 25), Sen CM+Noco (*n* = 18). Scale bar, 10 μm. **e.**-**g.** RMS traction stresses **e.** Maximum cumulative force **f.** and Net contractile moment **g.** of Sen CM and Sen CM+Noco exposed cells. **h.** Population-averaged mean squared displacement (MSD) as a function of time lag of T47D cells when exposed to Sen CM (red; *n* = 94), FM (black; *n* = 29), Sen CM+Noco (purple; *n* = 57), Sen CM+Taxol (yellow; *n* = 45). Red, purple, and yellow horizontal lines are the averaged sizes of T47D cell under each condition, and indicate that only cells exposed to Sen CM translocated more than their size over the 36 h of cell tracking. **i.** and **j.**. Averaged MSD evaluated at time lags of 15 min **i.** and 2 h **j.**. *n* = 94, *n* = 57, *n* = 45, respectively. ***P < 0.001 compared to cells exposed to Sen CM alone.

We next aimed to decipher the molecular mechanisms driving this unconventional mode of migration (Table 1). To study the contribution of microtubules to SASP-induced migration, cells were treated with microtubule-depolymerizing (nocodazole) and -stabilizing (taxol) agents. Treatment with nocodazole abrogated SASP-induced morphological changes and prevented the formation of cell tails (Figure 3a and 3b, Movie S3a-c). Cells not only failed to respond to Sen CM in the presence of nocodazole, but also retained the cytoskeletal organization of cells that remained round following SASP stimulation, with the formation of stress fibers in some cells (data not shown). Taxol-treated cells, however, did not form long cell trails, rather, they were shorter and less dynamic (Figure 3a and 3b; Movie S3d). Treatment with nocodazole additionally prevented the decrease in focal adhesions caused by Sen CM (Figure 2h, second panel), while taxol treatment did not enable any obvious change in focal adhesions when compared to Sen CM alone (Figure 3c). Treatment with nocodazole also prevented a SASP-induced decrease in cell traction and contractility and displayed contractile properties similar to cells that remained round in response to Sen CM (Figure 3d–3g, Figure 1j–1m). Both microtubule depolymerization and stabilization, however, significantly reduced cell migration in the presence of Sen CM (Figure 3h–3j).

### Actomyosin regulation of SASP-induced morphology and migration

**Figure 4:**
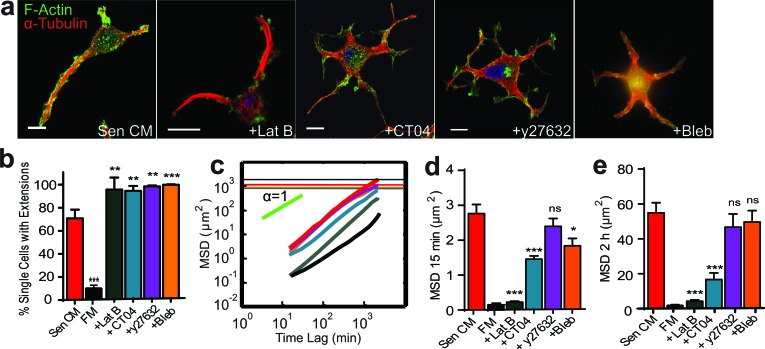
Role of RhoA/ROCK/myosin II in SASP-induced migration and morphology **a.** Fluorescence confocal micrographs of T47D cells after 48h exposure to Sen CM, Sen CM+250 nM Latrunculin B (LatB), Sen CM+25 μM Blebbistatin (Bleb) or Sen CM+2.0 μg/ml CT04 (Rho Inhibitor I), respectively, for 48h. Cells are stained for F-actin (green), α-tubulin (red) and nuclear DNA (blue). Scale bar, 10 μm. **b.** Percentage of single cells with extensions when exposed to Sen CM (red; *n* = 147), FM (black; *n* = 57), Sen CM+LatB (gray; *n* = 33), Sen CM+Bleb (orange; *n* = 101), Sen CM+y27632 (purple; *n* = 138) and Sen CM+CT04 (blue; *n* = 176) respectively after 48h. respectively. **c.** Population-averaged mean squared displacement (MSD) as a function of time lag when T47D cells are exposed to Sen CM (*n* = 94), FM (black; *n* = 29), Sen CM+LatB (*n* = 46), Sen CM+Bleb (*n* = 64), Sen CM+y27632 (*n* = 57), and Sen CM+CT04 (*n* = 45). Red, gray, orange, purple and blue horizontal lines are the averaged sizes of cells under each condition, and indicate cells that translocated more than their size over the 36 h of cell tracking. **d.** and **e.** Averaged MSD evaluated at time lags of 15 min **d.** and 2 h **e.**. *n* = 94, *n* = 46, *n* = 64, *n* = 57, *n* = 45 respectively. *P < 0.05, **P < 0.01 and ***P < 0.001.

In addition to microtubule dynamics, actin filament network remodeling plays a key role in cell polarization and migration. Members of the Rho family of GTPases are critical regulators of actin and microtubule cytoskeletal networks in response to extracellular signals [16, 22]. Recent work has demonstrated a functional crosstalk between microtubules and RhoA, a master regulator of actin filament organization, cell morphology, and cell contractility through the activation of downstream targets [23, 24]. These include Rho-associated coiled-coil kinase (ROCK), which acts as a regulator of myosin light chain (MLC) phosphorylation via phosphorylating and inhibiting myosin light chain phosphatase. To assess the role of F-actin and its regulators in the promotion of SASP-induced morphological and migratory changes, cells were treated with small-molecule inhibitors of the actomyosin network (Figure 4a). Surprisingly, when actin assembly was inhibited with Latrunculin B, SASP-stimulated cells generated more, but shorter extensions (Figure 4a). Similar responses were observed following the inhibition of Rho, ROCK and myosin II ATPase activity by CT04, Y27632, and blebbistatin respectively. We observed more extensions per cell; cells had > 4 extensions when treated with actin and Rho/ROCK/myosin inhibitors in the presence of Sen CM (Figure 4a). Moreover, these inhibitors promoted an increase in the number of cells with an elongated morphology and microtubule-rich extensions compared to Sen CM alone (Figure 4b).

These treatments, however, had differential effects on cell migration. Depolymerization of F-actin with Latrunculin B blocked cell migration regardless of the presence of cell extensions. However, CT04, Y27632, and blebbistatin treatment only induced a slight decrease in the displacement of cells (Figure 4c–4e and Movie S4 a-d). With CT04, Y27632, and blebbistatin treatment some cells displayed a “caged” pattern, where cellular motions are confined within cell extensions and failed to move beyond their own average size (Movie S4b).

### Rho and contractility inhibition is required to promote and maintain the SASP-induced phenotype

**Figure 5:**
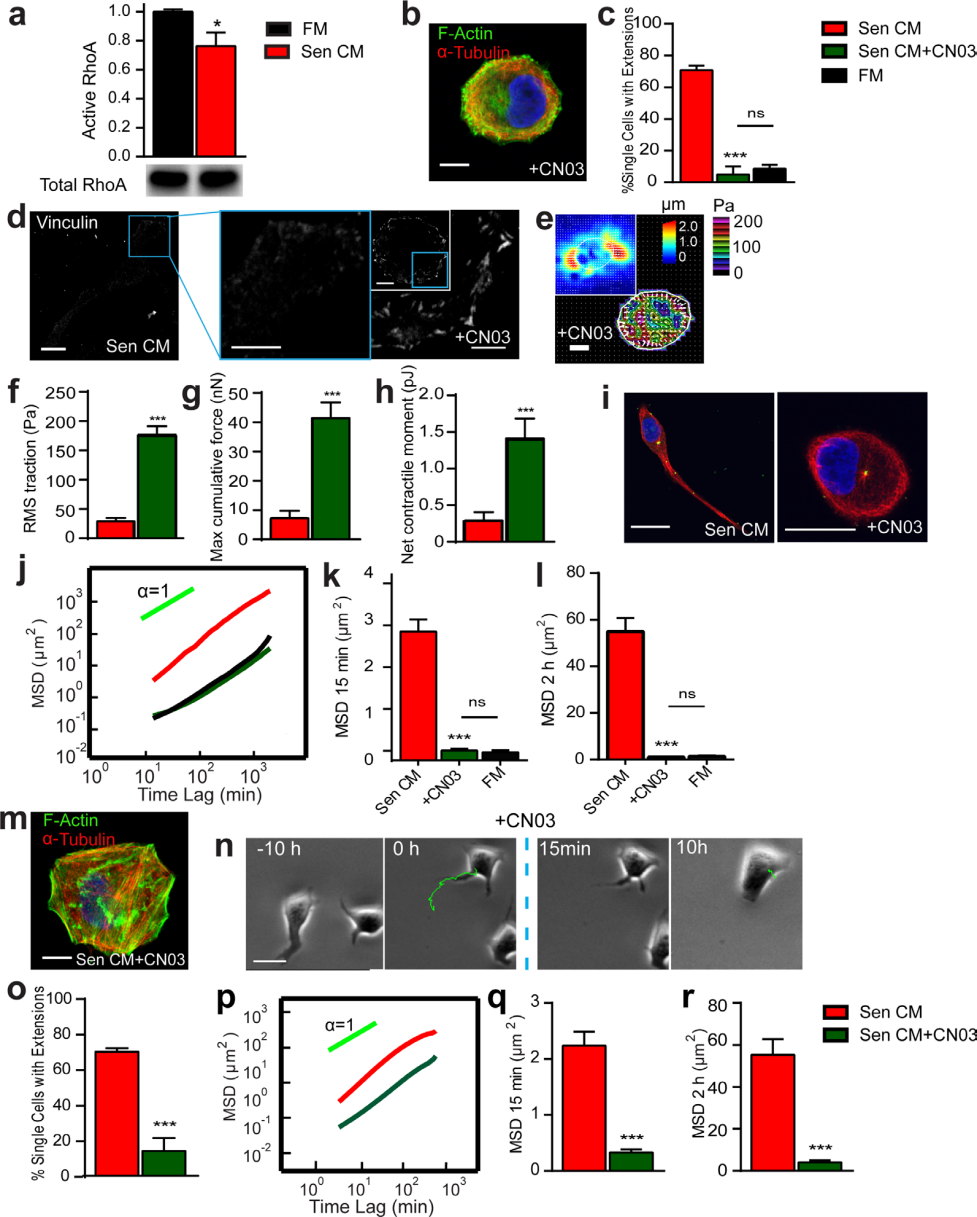
Rho inhibition is required to promote and maintain SASP-induced phenotype **a.** Rho activation levels of cells exposed to FM (black) and Sen CM (red). Activity levels were determined by GLISA add compared with total RhoA levels in total cell lysate **b.** Confocal micrographs of T47D cells after 48h exposure to Sen CM+ 1.0 μg/ml CNO3 (rho activator II). Cells were stained for F-actin (green), α-tubulin (red) and nuclear DNA (blue). Scale bar, 10 μm. **c.** Percentage of single cells with extensions when exposed to Sen CM (red; *n* = 147), Sen CM+CN03 (green; *n* = 38), FM (black; *n* = 57), respectively, for 48h. **d.** Fluorescence confocal micrographs of cells exposed to Sen CM and Sen CM+CN03 stained for vinculin, where blue boxes indicate zoomed region of T47D cells. Scale bar, 10 μm in original image and 5 μm in zoomed image. **e.** Bead displacement and traction field of T47D cells placed on a 1300 Pa compliant polyacrylamide gel and exposed to Sen CM+CN03. *n* = 20. Scale bar, 10 μm. (f-h) RMS traction stresses **j.** Maximum cumulative force **k.** and Net contractile moment **l.** of Sen CM and Sen CM+CN03 exposed cells. **i.** T47D cells immunostained for α-tubulin (red), pericentrin (green), and nuclear DNA (blue) after exposure to Sen CM (first panel) and Sen CM+CN03 (second panel) for 48h. Scale bar, 10 μm. **j.** Population-averaged mean squared displacement (MSD) as a function of time lag for T47D exposed to Sen CM (*n* = 94), Sen CM+CN03 (*n* = 28), FM (*n* = 29). (k and l). Averaged MSD evaluated at time lags of 15 min **k.** and 2 h **k.**. **m.** Confocal micrographs of T47D cells exposed to senescent CM for 48h then exposed to Sen CM then Sen CM+CNO3 for 24h. Cells were stained for F-Actin (green), α-tubulin (red) and nuclear DNA (blue). Scale bar, 10 μm. **n.** Phase-contrast micrograph of morphological and migratory phenotype of a representative cell 10h before and after CNO3 in the presence of SEN CM. The blue dotted line indicates when CN03 is added. **o.** % Single cells with extensions when exposed to Sen CM (red) for 48hr then Sen CM+CNO3 (green) 24h. *n* = 82. **p.** Population-averaged mean squared displacement (MSD) as a function of time lag of T47D cells when exposed to Sen CM before (red; *n* = 84) and after CN03 (green; *n* = 55). **q.** and **r.** Averaged MSD evaluated at time lags of 15 min **q.** and 2 h **r.**. *n* = 84 and *n* = 55 respectively. *P < 0.05, **P < 0.01 and *** *P* < 0.001.

Because the inhibition of Rho activity promoted an increase in the number of cell extensions, we next determined whether the reduction of Rho activity was involved in the promotion of an elongated, motile phenotype in the presence of Sen CM. The levels of activated RhoA (RhoA-GTP) were compared between cells exposed to serum-free fresh medium (FM) and Sen CM (Figure 5a, Figure S4a). Cells exposed to Sen CM showed decreased RhoA activity. Hence, we hypothesized that SASP-stimulated morphological and migratory phenotypes were mediated via RhoA GTPase inhibition. To assess this, cells were treated with CN03, a constitutive activator of RhoA, in the presence of Sen CM. Cells retained their round morphology (similar to nocodazole treatment) and failed to form cell extensions (Figure 5b and 5c). As shown in Figure 5b, cells treated with CN03 showed organized F-actin within the cell body while displayed disorganized microtubule (i.e. similar to cells treated with FM and/or cell that remained round upon Sen CM stimulation). Further, by constitutively active RhoA, only ∼5% of cells formed cell extensions and displayed insignificant differences in the percentage of cells with extensions when compared to cells exposed to FM alone (Figure 5c). Similar results were also obtained when T47D cells were transfected with constitutively active RhoA mutant and then exposed to Sen CM; cells expressing high amounts of the Myc-RhoA-Q63L remained round and displayed increased basal stress fibers (Figure S4, b-d).

Because cells treated with CN03 in the presence of Sen CM retained a similar morphology to round control cells, we next examined if constitutively activating RhoA would restore focal adhesion assembly in response to Sen CM. Cells displayed large focal adhesions along the cell cortex following RhoA activation (Figure 5d); however, vinculin staining revealed larger focal adhesions than control, round cells in the presence of Sen CM. Treatment with CN03 additionally prevented a SASP-mediated decrease in traction force and cell contractility and further enhanced it beyond the FM control (Figure 5e–5h). CN03 treatment also prevented changes in polarization seen with Sen CM (Figure 5i). Constitutively activating RhoA abrogated SASP-induced migration: the MSD of cells with activated Rho in the presence of Sen CM was similar to that of cells in FM (Figure 5j–5l).

We next asked whether RhoA inhibition was required to maintain SASP-induced morphology and migration (Figure 5n–5r). Cells were exposed to Sen CM for 48h to allow for morphological and migratory changes to occur, and then they were treated with CN03 for 24h in the continued presence of Sen CM. CN03-treated cells retracted their extensions and reverted back to a round morphology, with dense actin at the cortex and disorganized microtubules (Figure 5m). The percent of cells featuring trails decreased from ∼70% to ∼14% (Figure 5o). To observe this transition, cells were imaged for 24h with Sen CM alone followed by additional 24h after CN03treatment. Before treatment (10h), cells were still motile and featured long extensions; however, within a few minutes of CN03 treatment cells began to retract their tails and rapidly decreased migration concurrently (Figure 5n). Cell migration was further quantified between 24h pre and post treatment. Cells having constitutively active RhoA displayed significantly decreased migration regardless of SASP exposure (Figure 5p–5r). These results suggest that RhoA is critical for both the promotion and the maintenance of SASP-induced morphological, migratory, and force-generation changes.

### Inhibition of the Rho/ROCK/myosin II pathway recapitulates SASP-induced phenotype

**Figure 6:**
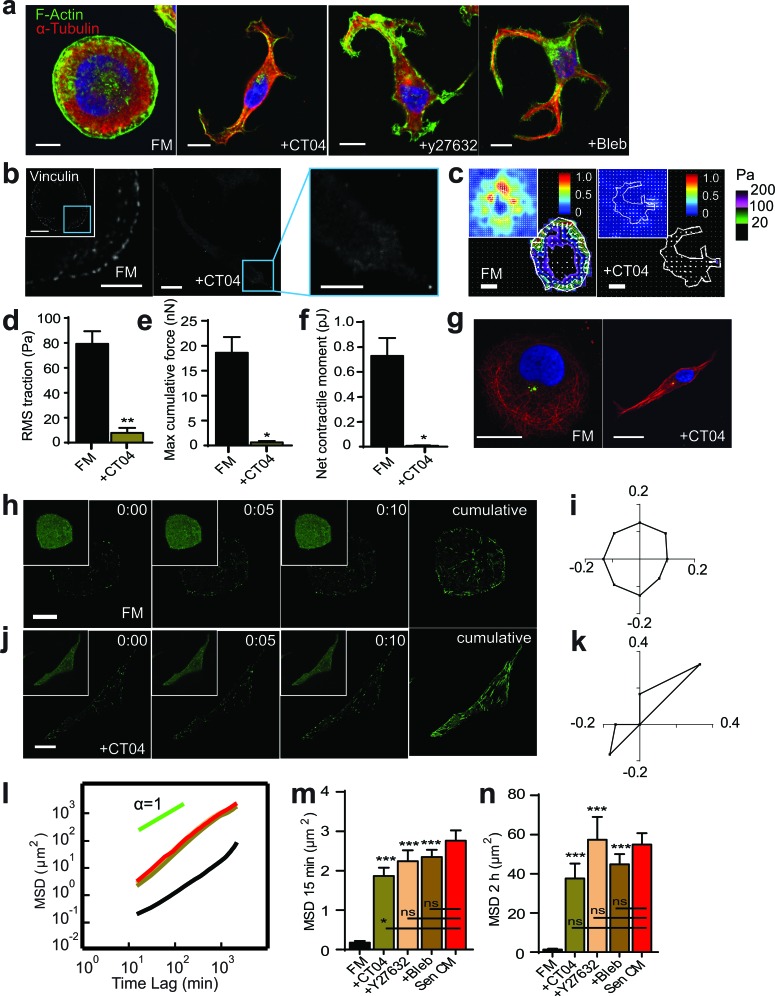
RhoA/ROCK/Myosin II inhibition recapitulates SASP-induced phenotype **a.** Fluorescence confocal micrographs of T47D cells after 48h exposure to FM, FM+25 μM Blebbistatin (Bleb) or FM+2.0 μg/ml CT04 respectively for 48h. Cells are stained for F-actin (green), α-tubulin (red) and nuclear DNA (blue). Scale bar, 10 μm. **b.** Fluorescence confocal micrographs of cells exposed to FM and FM+CT04 stained for vinculin, where blue boxes indicate zoomed region of T47D cells. Scale bar, 10 μm in original image and 5 μm in zoomed image. **c.** Bead displacement and traction field of T47D cells placed on a 1300 Pa compliant polyacrylamide gel and exposed to SF and SF+CT04. *n* = 25 and *n* = 5 respectively. Scale bar, 10 μm. (d-f) RMS traction stresses **d.** Maximum cumulative force **e.** and Net contractile moment **f.** of Sen CM and Sen CM+Noco exposed cells. **g.** T47D cells immunostained for α-tubulin (red), pericentrin (green), and nuclear DNA (blue) after exposure to FM (first panel) and FM+CT04 (second panel) for 48h. Scale bar, 10 μm. **h.**-**k.** T47D cells transfected with EB1-EGFP then exposed to FM and FM+CT04 for 24h are displayed every 5s for 10s for first 3 panels with a cumulative image shown in the last panel. Original images are displayed as inset per image. Scale bar, 10 μm. **i.** and **k.** Angular distributions of EB1-EGFP comets within FM **i.** and FM+CT04 **k.** cells within both cell center and periphery. *n* = 30 comets per cell. **l.**. Population-averaged mean squared displacement (MSD) cells as a function of time lag for T47D exposed to FM (black; *n* = 29), FM+Bleb (brown; *n* = 60), FM+y27632 (tan; *n* = 68), FM+CT04 (green) and Sen CM (red; *n* = 94). (n and o). Averaged MSD evaluated at time lags of 15 min **m.** and 2 h **n.**. *P < 0.05, **P < 0.01 and ***P < 0.001.

We asked whether the reduction of Rho alone was sufficient to promote and recapitulate SASP-induced morphological changes. Surprisingly, treatment with Rho inhibitor CT04 in FM promoted a similar formation of microtubule-rich cell extensions (Figure 6a). Cells transitioned from a round morphology and cytoskeletal organization, and adopted characteristics similar to those exposed to Sen CM alone. Similar results were also obtained when T47D cells were transfected with a dominant negative RhoA mutant and then exposed to FM (Figure S4, a-c).

Inhibition of either ROCK or myosin II activity resulted in similar cellular phenotypes. Cells that adopted an elongated phenotype with Rho inhibition significantly diminished focal adhesions like Sen CM exposed elongated cells (Figure 6b). Furthermore, Rho inhibition had a significant decrease in traction forces and contractility (Figure 6c–6f), down to values similar to those found in SASP-induced elongated cells (Figure 1,j-m). Rho inhibition also promoted an inverse, front-to-back polarization similar to SASP stimulation (Figure 6g, Figure S Figure 4e). Although cells treated with CT04 displayed similar microtubule organization to cells stimulated with Sen CM, we aimed to determine if End-Binding protein 1 (EB1) organization and directionality was recapitulated as well. Individual images collected 1s apart for 10s were overlay to display the cumulative directionality of EB1 comets. EB1 comets of cells in FM showed isotropic directionality (Figure 6h). Cells treated with CT04 alone, however, adopted polarized directionality (Figure 6j), where EB1 comets were enriched in the direction of cell tails. The directionality of actively growing EB1 comets, quantified by computing their time-dependent angular distributions, indicated that elongated cells had EB1 had polarized comets in the direction of the moving cell (Figure 6i and 6k).

Although morphology, traction forces, focal adhesion formation, and anisotropic EB1 dynamics were all recapitulated by direct Rho/ROCK/myosin II inhibition, we next wanted to assess whether migration was also similar to that of cells exposed to Sen CM. Analysis of MSDs showed that the inhibition of the Rho/ROCK/myosin II pathway in the absence of Sen CM was sufficient to recapitulate the migratory phenotype induced by Sen CM (Figure 6l–6n).

## DISCUSSION

Previous studies indicate that factors secreted by senescent stromal cells impose an aggressive behavior on neighboring cancer cells. This includes enhanced proliferation, increased tumor size, loss of cell-cell contacts, EMT, and invasion [5, 7–11]. Using non-invasive human breast carcinoma cell lines, we observed that factors secreted by senescent human fibroblasts additionally promote spontaneous morphological changes that are accompanied by migration. Elongated cells had extensions that were morphologically different from standard lamellipodial and filopodia protrusions, which are located at the front of the cell in conventional mesenchymal migration (Table 1) [25]. Rather, they were long trailing tails. Moreover, live-cell imaging revealed that, during migration, cells displayed a very thin or no lamellipodium and no apparent filopodial protrusions (Figure 1a and 1d). This morphological change provided cells with the ability to translocate. SASP stimulation decreases cell contractility, which in turn induces lower traction forces, inducing a gliding mode of cell migration.

In response to SASP, round non-motile cells spontaneously form microtubule-rich tails and actin-lacking cortex, along with a decrease in focal adhesions. Morphological changes are associated with the redistribution of the directionality of microtubule end binding protein EB1, as well as the promotion of an inverse, front-back polarity. In conventional mesenchymal migration, cells place their nucleus at the back and the MTOC at the front of the cell, a polarization that is tightly regulated by a myriad of proteins, including Cdc42 and PAR proteins [18, 26]. This inverse polarization has mainly been observed in migrating T cells and neutrophils [16, 22, 27], and not typically in mesenchymal or epithelial cells. Posterior MTOC position has however been seen with fibroblasts on constricted on fibrillar 1D patterns and 3D extracellular matrixes [28]. Similar inverse polarization was observed with Rho inhibition, suggesting a possible role of Rho in MTOC orientation. Changes in protein distribution and cell polarity may enable lateral movement in originally non-motile, non-polarized cells.

Microtubules are not believed to play “motor” function in conventional mesenchymal cell migration, but rather are required for establishing cell polarity [29]. Our results, however, demonstrate the integral role of microtubule assembly and dynamics in SASP-induced changes in cell morphology and onset of migration. Here, the inhibition of microtubule polymerization essentially prevented the formation of cell extensions and inhibited cell migration. Nocodazole treatment also prevented the decrease in focal adhesions and cell contractility. Microtubules sequester guanine nucleotide-exchange factors (GEF) that stimulate RhoA GTPase activation [22, 23, 30]. Depolymerization of microtubules in SASP stimulated cells may therefore enable RhoA activation, which may promote actin filament assembly. This may further prevent the depletion of the actin-rich cortex, the loss of focal adhesions and the decrease in cell contractility with Sen CM stimulation. Although microtubule stabilization also decreased cell movement, it did not prevent the formation of cell tails, but reduced their lengths and dynamics. Taxol treatment failed to induce a significant change in focal adhesion assembly when compared to SASP-induced elongated cells. This suggests the lack of involvement of microtubule dynamics in regulating SASP-induced morphological changes. These results collectively indicate that while microtubule integrity is required for driving the morphological transition from a round to an elongated phenotype, both its integrity and dynamics were essential for robust SASP-induced cell migration.

In typical migrating cells, RhoA mediates the formation of actin stress fibers, the generation of contractile forces required for cell tail retraction at the trailing edge, and the promotion of focal adhesion turnover [31] [32]. The depletion or inhibition of RhoA typically leads to a decrease in cell migration due to the inhibition of cell contraction via ROCK and MLC, or may increase migration by decreasing cell-substratum adhesion based on the cell type [33]. In our study, depletion of Rho isoforms and downstream targets in the presence of Sen CM promotes excessive formation of microtubule rich-cell extensions. These results, however, indicate that the presence of cell extensions is necessary but not sufficient for cell migration, where excessive tails did not further enhance migration, but either retained or reduced it in some conditions.

The excessive formation of cell extensions, however, provided evidence that Rho/ROCK/myosin inhibition may occur with SASP stimulation alone. This was investigated with RhoA G-LISA activation assay, which confirmed the decrease in RhoA-GTP in SASP stimulated cells when FM and Sen CM were compared. We observed that a 25% decrease in RhoA activity was sufficient to promote the phenotypic changes observed with Sen CM exposure. Not only was RhoA activity decreased, but myosin based contractility was also diminished with SASP stimulation

The constitutive activation of RhoA, which stimulates ROCK/myosin activity [16], prevented the loss of the actin rich cortex and prevented the decrease in focal adhesions, cell traction and myosin-mediated cell contractility. We further observed that with constitutive activation of RhoA, cells failed to promote or maintain migration in response to SASP. Furthermore, the inhibition of Rho and downstream targets, ROCK and myosin II recapitulate similar migratory and morphological changes as seen with SASP stimulation. Inhibition of Rho alone, which prevents ROCK/myosin II activity, also displayed a similar decrease focal adhesions and cell contractility as SASP stimulation. Rho inhibited cells also displayed similar polarization and microtubule organization when compared to SASP stimulated cells, with microtubule rich bundles at the cell tails and aligned directionality of EB1 comets in the polarized direction of the cell. We demonstrate that RhoA-GTP inhibition is both necessary and sufficient to initiate and maintain morphological and migratory changes in response to SASP stimulation.

It is possible that SASP promotes the breakdown of the actin-rich cortex, via RhoA/ROCK inhibition, enabling microtubule expansion and bundling, whereby promoting the formation of cell tails. Although the overall cell morphology seems to play an integral role in SASP-induced cell migration, cells still require F-actin and microtubule assembly and dynamics to translocate significantly. However, in contrast to conventional mesenchymal migration, SASP stimulation promoted enhanced cell migration via contractility and focal adhesion inhibition. This may suggest a possible gliding mechanism (Table 1), where SASP-induced migration may be promoted in part by decreasing cell-substratum adhesion [33]. This behavior is similar to that of neutrophils, which display a rapid migratory phenotype while lacking focal adhesions [33]. These results collectively suggest the crosstalk of actin and microtubules in the promotion of SASP-induced migratory and morphological changes.

## MATERIALS AND METHODS

### Cell culture

T47D and MCF 7 cells (ATCC) were cultured in RPMI 1640 medium (ATCC) and DMEM respectively supplemented with 10% (v/v) fetal bovine serum (Hyclone Standard) and 1% (v/v) Penicillin/streptomycin (Sigma). Cells were passaged every 3-4 days for a maximum of 10 passages. Wi-38, BJ and IMR-90 cells (ATCC) were cultured in Eagle's Minimal Essential Medium (EMEM; ATCC) supplemented with 10% (v/v) fetal bovine serum (Hyclone Standard) and 1% (v/v) penicillin/streptomycin (Sigma). Cells were passaged every 2-3 days. Human fibroblasts were rendered senescent by treatment with 50 μM bleomycin sulfate (Enzo Life Sciences) for 4 h and allowed to recover for 8 days. Cells were verified to be senescent via Ki-67 immunostaning and a count of population doublings over a period of 4 days. All cells were maintained at 37°C and 5% CO_2_ in a humidified environment during culture and imaging. T47D cells were seeded unto tissue culture dishes and allowed to adhere and spread for a 24h. Cells were then washed 3x with PBS and incubated with EMEM based FM or CM.

### Drug treatments

The microtubule destabilizer nocodazole (Sigma), the microtubule stabilizer taxol (also known as paclitaxel; Invitrogen), the F-actin disassembly drug latrunculin B (Sigma), the non-muscle myosin II inhibitor blebbistatin (Sigma), the ROCK inihibitor, Y27632 (Sigma), the Rho inhibitor CT04 (Cytoskeleton Inc.), and the Rho activator CN03 (Cytoskeleton Inc.) were diluted from stock using culture medium. Nocodazole was used at a final concentration of 0.5 μg/ml; taxol was used at a final concentration of 1 μM. Latrunculin B was used at a final concentration of 250 nM; blebbistatin was used at a final concentration of 25 mM; Y27632 was used at a final concentration of 15 mM; CT04 was used at a final concentration of 2 μg/ml; CN03 was used at a final concentration of 1 μg/ml.

### Conditioned medium and Elisa assay

Conditioned medium (CM) from human fibroblasts was prepared by washing cells 3x with PBS and incubating cells with serum-free EMEM for a period of 24 h. Elisa kits from R&D Systems were used to detect IL-6 (D6050) and IL-8 (D8000C) according to instructions.

### Cell tracking

Cells were exposed to FM or CM conditions for 24h before live cell experiments. Cells were imaged with a Nikon TE2000 microscope with a phase contrast 10x objective every 15 min for 36 h. Cells were tracked using Metamorph (Molecular Devises Corp) software to track the dependent centroids of individual cells. Individual cells were tracked using the Metamorph imaging software (Molecular Devices). A custom MATLAB program (MathWorks) calculated the MSD for each cell using the *x* and *y* coordinates of the cell centroid using the following equation: MSD = < [*x*(*t+*Δ*t*)-*x*(*t*)]^2^+[*y*(*t+*Δ*t*)-*y*(*t*)]^2^ > [34].

### Immunofluorescence

To visualize Ki-67, microtubules, F-actin, and vinculin, cells were fixed in 4% paraformaldehyde (Electronic Microscopy Systems) for 10 min and permeablized with 0.1%Triton X-100 (Fisher Chemicals) for 10 min. Cells were blocked at room temperature for 1 h in 1% BSA (Sigma). Subsequently, cells were incubated in primary antibodies for 1 h at room temperature. Antibodies used included: Rb. Anti-Ki-67 (1:50; ab16667 Abcam), Ms. Anti-Tubulin (1:1000; ab Abcam), Ms. Anti-Vinculin (1:400; Sigma). Cells were then incubated with either Alexa 568 conjugated donkey anti-mouse or anti-rabbit secondary antibodies, Hoeccht 33342 and phalloidin for 1h at room temperature, after which cells were washed with PBS. Fluorescent images were collected after 48 h exposure to FM or CM conditions. Cells were imaged using a Nikon A1 confocal microscope using a ×60 water-immersion lens.

### Actin-rich edges

The boundaries of randomly selected cells stained for F-actin were traced using NIS-Elements image analysis software (Nikon). The percent of actin rich edges were calculated by dividing the length of the cell containing a dense F-actin network by the total circumference of the cell [35].

### RhoA activation and immunoblotting

T47D cells were plated and incubated with FM or CM for 48 h. RhoA G-LISA kit from Cytoskeleton Inc. was used to assess RhoA activity according to instructions. Total RhoA blots utilized the same lysates used in the G-LISA assay. Proteins were resolved on 12% precast polyacrylamide (Bio-Rad). Blots were probed with Rb-Anti RhoA antibody (Cell Signaling) followed by Rabbit HRP antibody (Cell Signalling). Immunoblots were visualized with ECL clarity and Image lab (Bio-Rad). Densitometry measurements were performed with Image Lab software (Bio-Rad).

### Fourier transform traction microscopy

An elastic gel block (Young's modulus ∼1300 Pa, Poisson's ratio 0.48) was made of acrylamide (2%), bis-acrylamide (0.25%) and red fluorescent latex beads (diameter 0.2 μm, 1:100 dilution by volume). A droplet of the solution was added to an activated glass-bottom MatTek (P35G-0-14-C) dish, covered with a circular coverslip (12mm in diameter), and left to polymerize (upside down) for 45 min. After polymerization, the coverslip was removed and the surface of gel block was coated with type I collagen (0.2 mg/ml in PBS) using photo-reactive crosslinker, Sulfo-SANPAH (Pierce). Gels were typically 50-70 μm thick. T47D cells were plated onto polyacrylamide gels and allowed to spread and stabilize for 24h. FM and CM were then added for 24h. Phase-contrast images of the cells and fluorescent images of the beads by 470-nm ultraviolet illumination were collected. To assess the displacement of beads in the relaxed gel, cells were detached from the substrate with 0.5% trypsin/EDTA.

### Tracking EB1-EGFP comets

EB1-EGFP plasmid was transfected into T47D cells using Lipofectamine 3000 (Life technologies) according to product specifications. The medium was changed to the condition being assessed for 24h. Cells were imaged on a Nikon A1 confocal at a rate of 1 frame/s, using a 60X oil-immersion objective lens. EB1-GFP dynamics was analyzed using plus-end tracking module in the u-track software package [36, 37].

### RhoA mutants

T47D cells were transfected using Lipofectamine 3000 (Life technologies) with either myc-RhoA-Q63L then exposed to Sen CM or myc-RhoA-T19L then exposed to FM for 48h. Cells were fixed and deteched with Ms anti-Myc antibody (Proteintech). Cells were then imaged with a Nikon TE300 and Nikon A1 confocal. Imaged were further scored for myc positive or negative cells.

### 3D Spheroid Assay

T47D cells were Trypsinized and resuspended in EMEM serum free media. Cells were mixed with EMEM sf and methacott at a (3:1) mixture to obtain a final cell concentration of 100k/ml. 100 μl of medium/methacott mixture was added to each well of a 96-well round bottom non-adhesive dish. Cells were then spun at 1500 rpm for 20 min and stored in a 37-degrees incubator for four days to allow spheroids to form. Medium was then removed and 1mg/ml collagen I containing FM or Sen CM was then added on top of spheroids and allowed to gel for 1 hr at 37 degrees. 200 μl of FM or Sen CM was then added on top of gel. Cells were imaged 24 h later. Maximum radial distance was measured by determining the spheroid center and measuring the distance traveled by the maximal displaced cell. These values are normalized to the initial spheroid conditions (Day 0)

### Statistics

The number of cells and biological repeats (n) for each experiment are indicated in the figure captions. Mean values ± s.e.m. and statistical analyses were calculated and plotted using Graphpad Prism (Graphpad Software, San Diego, CA). One way ANOVA and unpaired *t*-tests were conducted to determine significance, depending on the number of variables compared. Significance (*p*) was indicated within the figures using the following scale: *** for *p* < 0.001, ** for *p* < 0.01, and * for *p* < 0.05.

## SUPPLEMENTARY MATERIAL FIGURE AND MOVIES














