# Supplementary material for: Senescent stromal cells induce cancer cell migration via inhibition of RhoA/ROCK/myosin-based cell contractility
Source: Oncotarget. 2015 Oct 1;6(31):30516–31. doi: 10.18632/oncotarget.5854 (PMC4741548; doi:10.18632/oncotarget.5854)
Supplement: Supplementary file 1 [file oncotarget-06-30516-s001.pdf]

Senescent stromal cells induce cancer cell migration via inhibition of RhoA/ROCK/myosin-based cell contractility

Supplementary Material

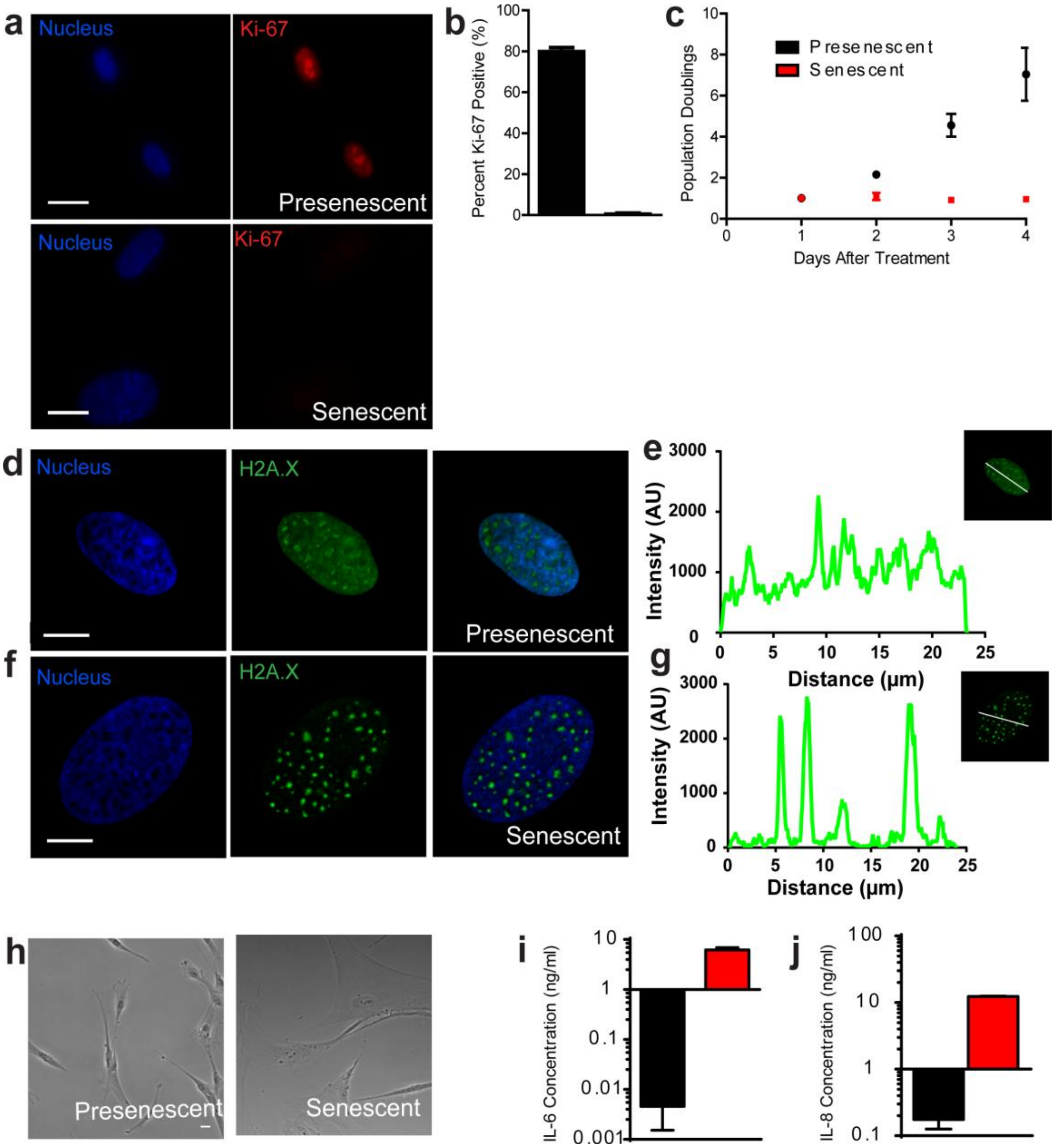

Figure S1

(a) Fluorescence confocal micrographs of WI-38 fibroblasts cells stained for KI-67 (red) and nuclear DNA (blue) 8 days after treatment with bleomycin or vehicle. Scale bar, 20 µm. **(b)** Percentage of cells that are KI-67 positive **(c)** Population doublings assessed after

treatment with bleomyin and vehicle up to four days after treatment. **(d-g)** Fluorescence confocal micrographs of WI-38 fibroblasts cells stained for H2A.X (green) and nuclear DNA (blue) 8 days after treatment with bleomycin or vehicle. (**e and g**) Intensity profiles of panel (d and f). **(h)** Phase micrographs of WI-38 fibroblasts 8 days after treatment with bleomycin or vehicle. **(i-j)** IL-8 and IL-6 concentrations in Sen CM.

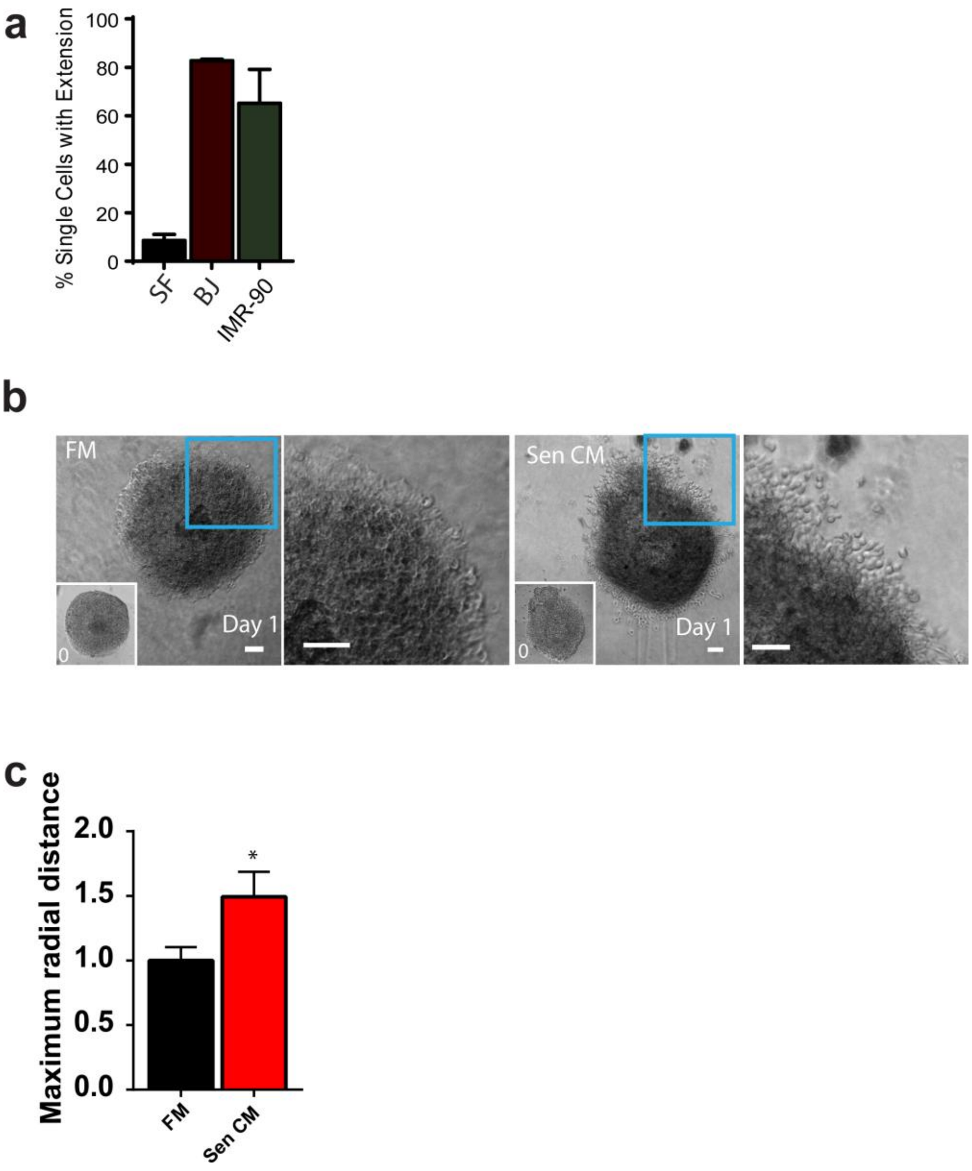

Figure S2

(a) Percentage of single cells with extensions when exposed to Fresh media (FM) (n=57), Sen CM from BJ fibroblasts (n=165) and Sen CM from IMR-90 fibroblasts (n=63), respectively after 48h. **(b)** T47D spheroids embedded in collagen after initial (Day 0) and 24h (Day 1) exposure to FM or Sen CM. White insets are Day 0 (initial) conditions. Blue squares are the enlarged regions in the panel following panel. **(c)** Maximum cells displacement from the radius of each cluster to the cluster periphery after 24 h exposure to either FM and Sen CM respectively. n=10, n=8 respectively.

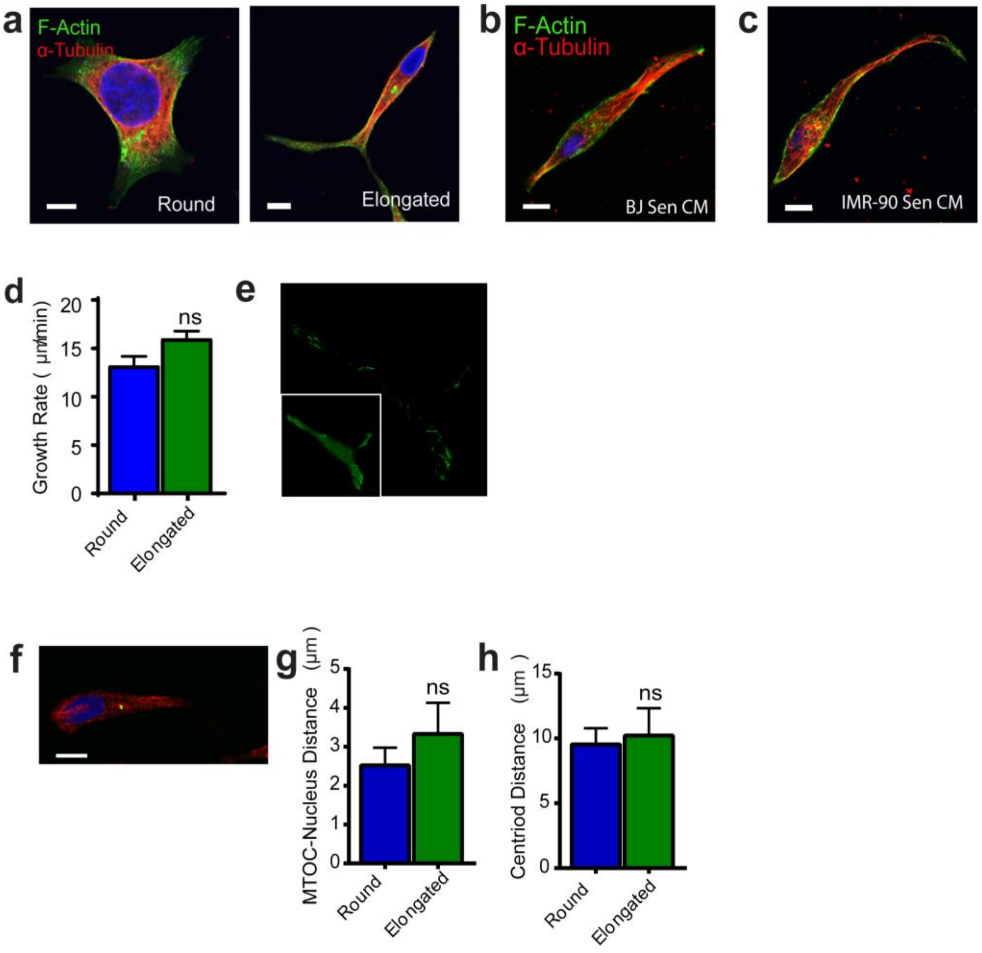

Figure S3

MCF7 cells stained for F-actin (green), α-tubulin (red) and nuclear DNA (blue) after exposure to Sen CM for 48h. **(b-c)** Fluorescence confocal micrographs of T47D cells after 48h exposure to Sen CM from BJ fibroblasts (b) and IMR-90 fibroblasts (c). **(d)** Round and elongated T47D cells- transfected with EB1-EGFP then exposed to Sen CM for 24h and tracked for growth rate (speed) of EB1 comets **(e)** Cumulative image of 10 consecutive time frames for 10s with original images displayed as insets. **(f)** T47D cell immunostained for α-tubulin (red), pericentrin (green), and nuclear DNA (blue) after exposure to Sen CM. Scale bar, 10 μm **(g-h**) MTOC and nucleus distance (shortest distance between nucleus edge and MTOC centroid) (g) and centroid distance (the distance between the nuclear centroid and MTOC centroid) (h).

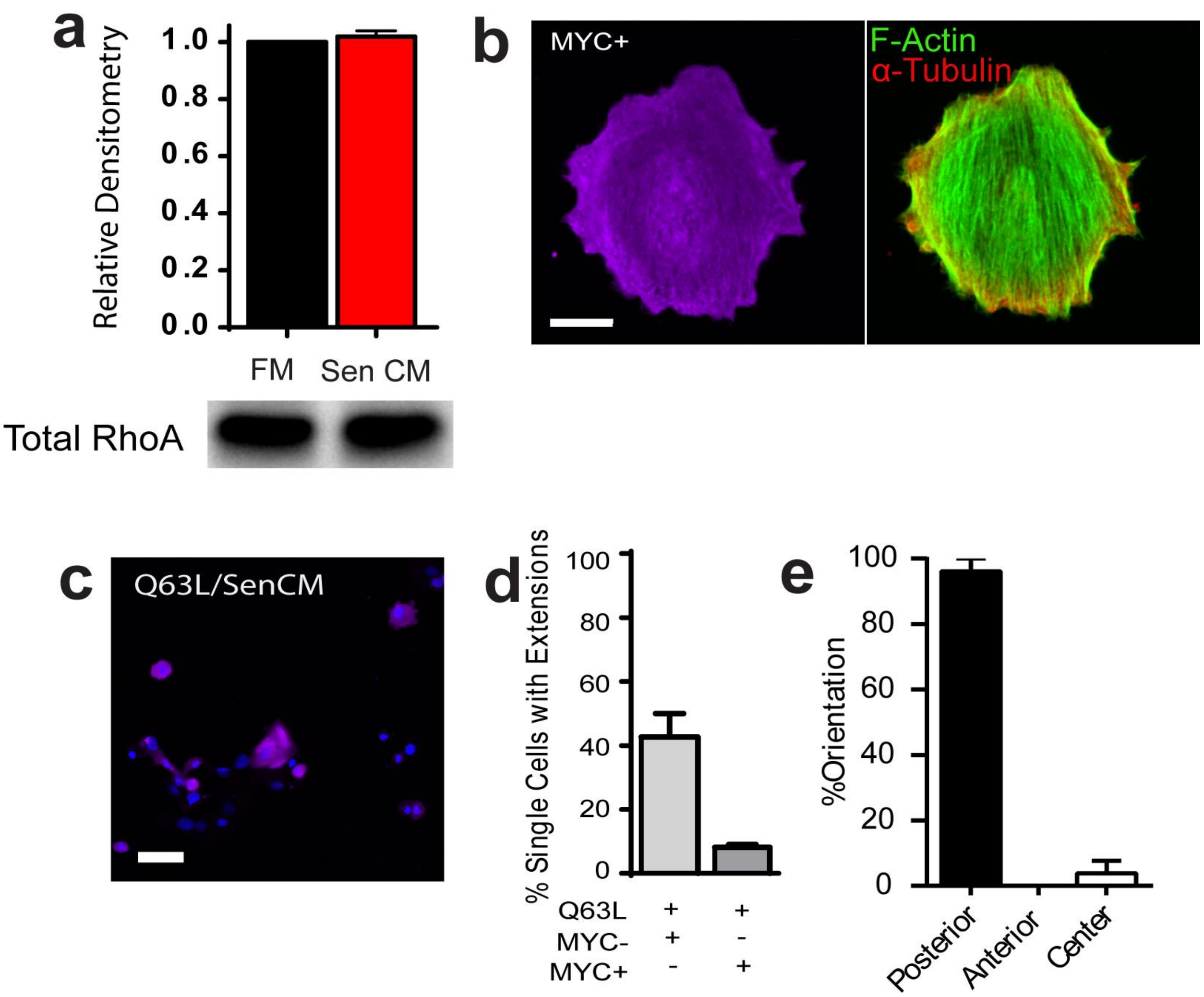

Figure S4

(a) Total RhoA blot of T47D cells exposed to FM and Sen CM. **(b-d)** T47D cells- transfected with myc-RhoA-Q63L then exposed to Sen CM for 48h. (b) Confocal micrograph of a myc positiveT47D cell stained for F-actin (green) and α-tubulin (red) after exposure to Sen CM for 48h. (c) Low magnification images of transfected cells immunostained for myc. Brightly stained cells indicate myc (mutant) expression. Percent single cells with extensions expressing high (myc+) and low (myc-) levels of myc.

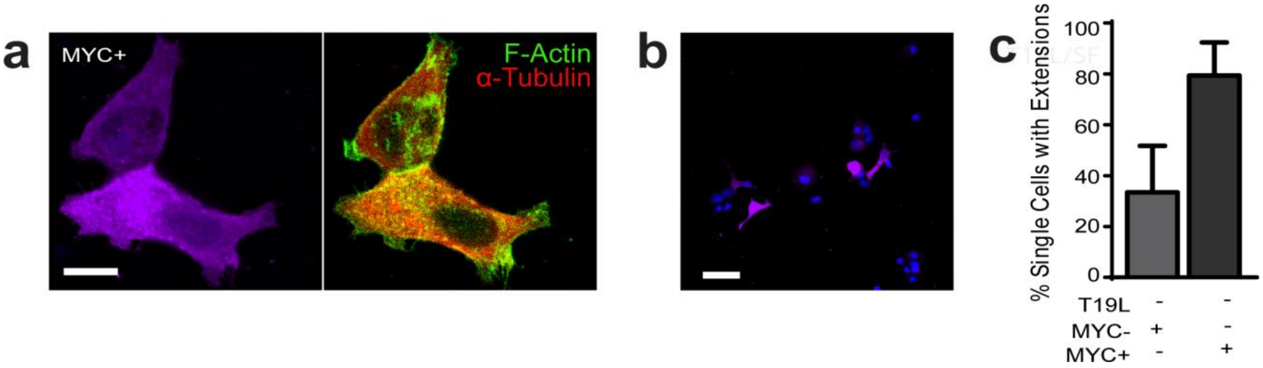

Figure S5

(a-c) T47D cells transfected with myc-RhoA-T19L then exposed to FM for 48h. (a) Confocal micrograph of a myc positiveT47D cell stained for F-actin (green) and α-tubulin (red) after exposure to FM for 48h. (b) Low magnification images of transfected cells immunostained for myc. Brightly stained cells indicated myc (mutant) expression. (c) Percent single cells with extensions expressing high (myc+) and low (myc-) levels of myc.

Movie S1

Low magnification movie of T47D cells under FM (a) and Sen CM (b) conditions. Typical movie (21.5 h) of a T47D cell undergoing change in morphology induced by Sen CM (c).

Movie S2

Round (a) and elongated (b,c) T47D cells- transfected with EB1-EGFP then exposed to Sen CM for 24h then imaged in 1s intervals for 3 minutes.

Movie S3

Movie (36 h) of a typical T47D cell under Sen CM (a), FM (b), Sen CM+ Noco (c), Sen+Taxol (d) conditions.

Movie S4

Typical movie (36 h) of a T47D cells under Sen CM+LatB (a), Sen CM+CT04 (b), Sen CM+Y27632 (c), Sen CM+Blebbistatin (d) conditions.

Movie S5

Typical movie (36 h) of a T47D cell under Sen CM + CN03 conditions.

Movie S6

Typical movie (36 h) of a T47D cell under FM + CT04 conditions.
